# *C. elegans* ATG-5 mutants associated with ataxia

**DOI:** 10.17912/micropub.biology.000792

**Published:** 2023-06-02

**Authors:** Azusa Yugeta, Hiroki Arai, Daiki Takahashi, Nami Haruta, Asako Sugimoto, Hirokazu Arimoto

**Affiliations:** 1 Life Sciences, Tohoku University, Sendai, Miyagi, Japan

## Abstract

Intercellular cleaning via autophagy is crucial for maintaining cellular homeostasis, and impaired autophagy has been associated with the accumulation of protein aggregates that can contribute to neurological diseases. Specifically, the loss-of-function mutation in the human autophagy-related gene 5 (ATG5) at E122D has been linked to the pathogenesis of spinocerebellar ataxia in humans. In this study, we generated two homozygous *C. elegans* strains with mutations (E121D and E121A) at positions corresponding to the human ATG5 ataxia mutation to investigate the effects of ATG5 mutations on autophagy and motility. Our results showed that both mutants exhibited a reduction in autophagy activity and impaired motility, suggesting that the conserved mechanism of autophagy-mediated regulation of motility extends from *C. elegans* to humans.

**Figure 1.**
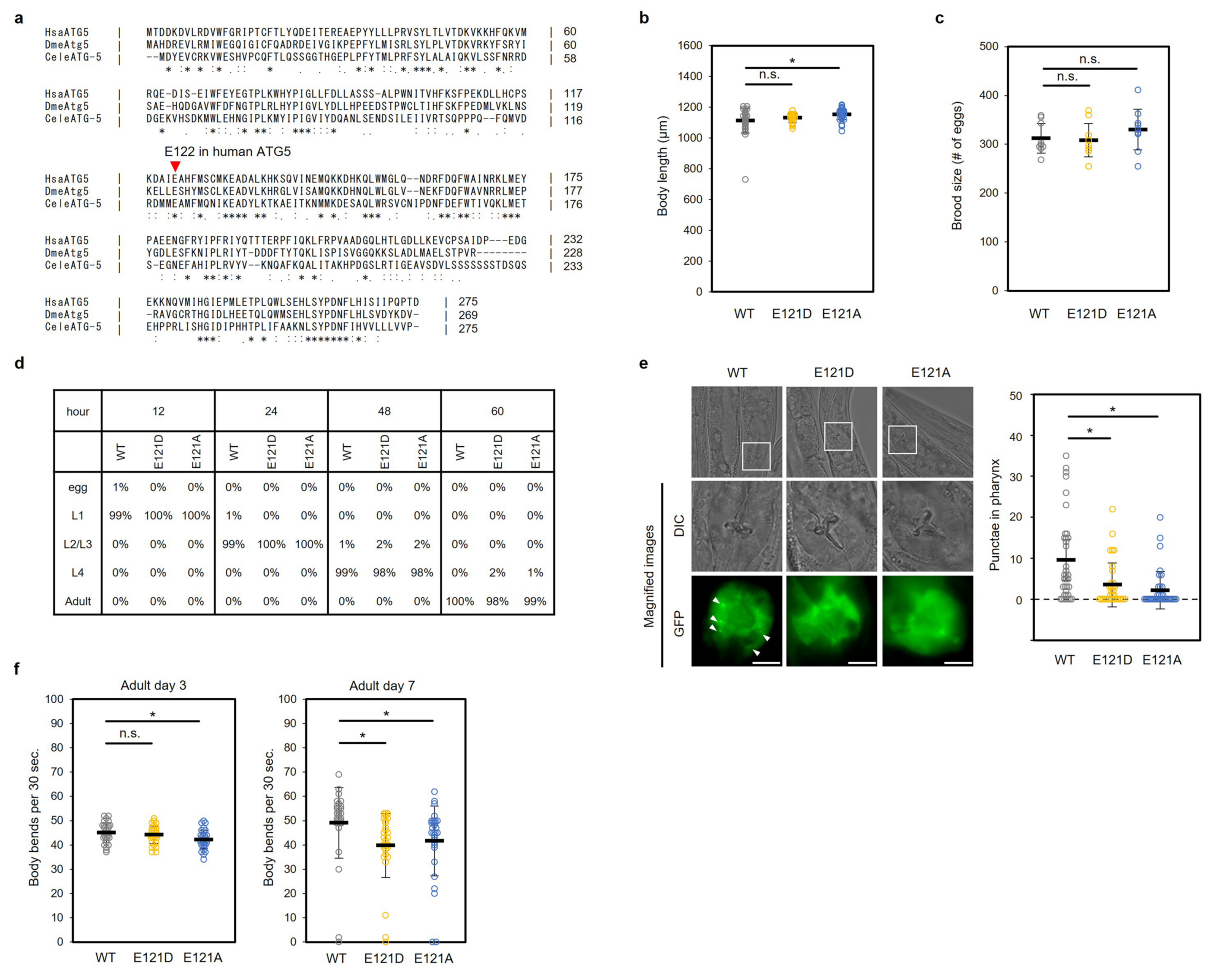
(a) Amino acid sequence alignment among ATG5 orthologs from human (HsaATG5), *Drosophila melanogaster* (DmeAtg5) and *Caenorhabditis elegans* (CeleATG-5) using CLUSTALW. The red arrowhead indicates E122 in human ATG5. (b) Body length was measured in wild-type (N2) (N=30), *atg-5(E121D)* (N=28) and *atg-5(E121A)* (N=40) adult day 1 animals. Data from the all analyzed worms are plotted. Horizontal lines and error bars indicate means ± s.d. (c) Brood size of wild-type (N2) (N=9), *atg-5(E121D)* (N=9) and *atg-5(E121A)* (N=9) animals was determined by counting the number of fertilized eggs and offspring throughout their lifetime. Plots show the number of fertilized eggs per worm. Horizontal lines and error bars indicate means ± s.d. (d) Growth rate was analyzed in wild-type (N2), *atg-5(E121D)* and *atg-5(E121A)* animals. Approximately 100 eggs (WT (N=96), E121D (N=97), E121A (N=102)) were collected and the number of worms at each stage (egg, L1, L2−L3, L4, adult) was counted every 12 hours. (e) Autophagy flux was measured in wild-type (WT) (N=36), *atg-5(E121D)* (N=33), and *atg-5(E121A)* (N=37) animals expressing *lgg-1p::gfp::lgg-1* . Worms at the L4 stage were starved for 8 h to induce autophagy. The white arrowheads indicate LGG-1 punctae. Plots show the number of GFP::LGG-1 punctae in the pharynx. Horizontal lines and error bars indicate means ± s.d. Scale bar, 10 µm. (f) The frequency of body bends on adult days 3 and 7 in WT, *atg-5(E121D)* and *atg-5(E121A)* (N=30) animals. Plots show the frequencies of body bends. Horizontal lines and error bars indicate means ± s.d. n.s., not significant, *P < 0.05 by Dunnett’s test.

## Description

Autophagy is an intracellular degradation system that operates constitutively. This system selectively degrades abnormal intracellular molecules, such as aggregated proteins and impaired organelles, in response to their production (Yamamoto et al., 2023) . Animals deficient in autophagy-related genes (ATGs), which are required to drive autophagy, are affected by neurodegenerative diseases with accumulation of abnormal proteins (Hara et al., 2006) . These indicate that intracellular cleaning via autophagy contributes to the suppression of disease development through the maintenance of homeostasis.

The autophagy pathway is based on large-scale membrane trafficking, particularly the generation and elongation of isolation membranes that sequester substrates. ATG5 is one of the most well-known ATGs involved in sequestration membrane elongation. Specifically, ATG5 catalyzes the ATG8 lipidation reaction through covalent binding with ATG12 for the recruitment of ATG8 on the isolation membrane (Mizushima et al., 2011) . Note that ATG5 is functionally and structurally conserved from humans to *C. elegans* ( Fig. 1a ). A pathogenic ATG5 E122D mutation leading to human spinocerebellar ataxia reduces both the interaction with ATG12 and autophagy induction (Kim et al., 2016) . However, there are insufficient studies on the pathogenesis of spinocerebellar ataxia due to a defect in the autophagy machinery. To address this issue, we generated a homozygous nematode strain with an *atg-5* mutation at a position corresponding to a human ataxia mutation ( *atg-5 (tj130[E121D])* ). We also generated an *atg-5* mutant in which Glu121 was converted to alanine ( *atg-5 (tj122[E121A])* ) to investigate the importance of carboxylate side chain of the glutamate residue. The side chain of alanine, the methyl group, is sterically small and does not engage in hydrogen bonding or ionic interactions. In both *atg-5* mutants, no marked differences in body length, brood size, and growth rate from wild-type worms were observed ( Fig. 1b −d).

To observe autophagy levels in these mutants, we utilized GFP-tagged LGG-1 (GFP:: LGG-1 ), a *C. elegans* ATG8 ortholog, that is widely accepted as an autophagosome marker (Mizushima, 2004) . In worms in which autophagy is induced, GFP:: LGG-1 is detected as dot-like structures in cells (Kang et al., 2007) . In both *atg-5* mutants, the number of GFP:: LGG-1 dots in the pharynx was lower than wild-type worms, suggesting a permanent decrease in autophagy ( Fig. 1e ).

A marked decrease in locomotion ability has been observed in patients with spinocerebellar ataxia (Kim et al., 2016) . We tested whether a similar phenotype was observed in *atg-5 (tj130[E121D])* or *atg-5 (tj122[E121A])* , using body bend frequency to evaluate the locomotion ability of worms (Onken & Driscoll, 2010) . At day 7 of adulthood, both *atg-5(E121D)* and *atg-5(E121A)* worms showed a statistically significant reduction in body bend frequency ( Fig. 1f ). As early as day 3 of adulthood, a tendency of reduced frequency of body bend was observed in the *atg-5(E121A)* mutants ( Fig. 1f ). It is interesting because, to our knowledge, such alanine mutant has not been reported in human ataxia. Collectively, these results indicate that the E121 mutation on ATG-5 reduced the locomotion ability of worms that might be related to the human spinocerebellar ataxia.

In summary, we introduced an ATG5 mutation associated with human spinocerebellar ataxia into *C. elegans* for the first time and demonstrated that this mutation causes locomotor defects in the nematode. Our analysis of GFP:: LGG-1 dots indicated that the two mutant strains created in this study had defective autophagy activity, but their impact on locomotion appeared to be somewhat different. This nematode model could be used to investigate the etiology and pathogenesis of spinocerebellar degeneration in humans.

## Methods

**Establishment the ATG-5 mutants by CRISPR/Cas9**

The *atg-5* mutants, SA1525{ *atg-5 (tj130[E121D])* } and SA1498{ *atg-5 (tj122[E121A])* } were generated by the CRISPR/Cas9 genome editing system. The sgRNA and the single-strand oligodeoxynucleotide (ssODN) template (both shown in Reagents table) were injected into wild-type N2 hermaphrodites with purified Cas9 protein (Sugimoto lab, Tohoku University, Sendai) and injection markers ( *dpy-10* sgRNA and ssODN) using the same procedure as described by Arribere *et al.* The F1 progeny showing a dumpy phenotype from the P0 injected worms were isolated. These F1 worms were genotyped to confirm the target mutations using the following primers: 5’-ggcaaaaaattgatatttgtaggtgctcag-3’ and 5’-gtttgcaatagtatgggagttttctgactg-3’. PCR products were treated with the restriction enzyme A *fl* II and the digested samples were sequenced. After confirming the mutation, the mutant strains were outcrossed with N2 and removed *dpy-10* mutation.

**Body length**

L4 larvae were collected, and 16-18 hours later, animals were taken under a microscope and measured as 1-day adults. At least 28 worms were analyzed.

**Brood size**

Individual L4 stage worms (N=9) were transferred every 12 hours. The number of fertilized eggs and hatched larvae were counted repeatedly until the eggs were laid unfertilized.

**Growth rate**

After performing timed-egg-lay of 10 adult worms at 20°C, collecting approximately 100 eggs, the number of worms at each stage (egg, L1, L2−L3, L4, adult) was counted every 12 hours.

**Autophagy monitoring assay**

Autophagy was monitored by observing the formation of GFP-tagged LGG-1 /Atg8 punctae in pharynx of strains DA2123 ( *adIs2122 [ lgg-1 p::GFP:: lgg-1 ]* ), SA1530 ( *adIs2122 ;tj130[E121D]* ) and SA1514 ( *adIs2122 ;tj122[E121A]* ). Worms at the L4 stage were randomly selected, and the formation of GFP-tagged LGG-1 punctae in the pharynx of at least 33 worms was analyzed. Images were acquired using fluorescence microscopy, BZ9000, Keyence.

**Measurement of frequency of body bends**

The assay was performed as previously described (Onken & Driscoll, 2010) . Thirty worms in 20 µL of M9 buffer were transferred to a glass slide. After 2 minutes, the behavior of the worms in the M9 buffer was recorded for 30 seconds. Body bends were counted by reviewing the 30−second movies (N=30).

**Statistical analysis**

Statistical analyses were performed using Dunnett’s test. In all tests, *P* -value of < 0.05 was considered statistically significant.

## Reagents

**Table:** 

**sgRNA (in vitro transcription)**		
5’-actatttgaagactaaagcc-3’		
**ssODN**		
ATG-5 (E121D)	gagattattgtacgaacatcccagccgcccccgcaatttcaaatggtCgaCcgggatatgatggaTgcaatgtttatgcaaaatattaaggaagccg	
ATG-5 (E121A)	gagattattgtacgaacatcccagccgcccccgcaatttcaaatggtCgaCcgggatatgatggCTgcaatgtttatgcaaaatattaaggaagccg	
***C. elegans* strains **		
Strain	Genotype	Available from
SA1525	*atg-5 [tj130(E121D)] *	This work
SA1498	*atg-5 [tj122(E121A)] *	This work
DA2123	*adIs2122 [ lgg-1 p::GFP:: lgg-1 + rol-6 ( su1006 )] *	CGC
SA1530	* tj130[ atg-5 (E121D)]; adIs2122 [ lgg-1 p::GFP:: lgg-1 + rol-6 ( su1006 )] *	This work
SA1514	* tj122[ atg-5 (E121A)]; adIs2122 [ lgg-1 p::GFP:: lgg-1 + rol-6 ( su1006 )] *	This work
